# Improving a scissor‐action couch for conformal arc radiotherapy and radiosurgery

**DOI:** 10.1120/jacmp.v5i3.2026

**Published:** 2004-10-21

**Authors:** Kaile Li, Cedric X. Yu, Lijun Ma

**Affiliations:** ^1^ Department of Radiation Oncology University of Maryland School of Medicine 22 South Greene Street Baltimore Maryland 21201 U.S.A.

**Keywords:** conformal arc, couch, isocenter alignment

## Abstract

We have developed a method to improve the setup accuracy of a Varian Clinac 6/100 couch for delivering conformal arc therapy using a tertiary micro multileaf collimator (MLC) system. Several immobilization devices have been developed to improve the mechanical stability and isocenter alignment of the couch: turn‐knob harnesses, double‐track alignment plates, and a drop‐in rod that attaches the couch to the concrete floor. These add‐on components minimize the intercomponent motion of the couch's scissor elevator, which allows consistent treatment setup. The accuracy of our isocenter couch alignment is an improvement over the above devices, within 1 mm of their accuracy. The couch has been used with over 15 patients and with over 50 modulated conformal arc treatment deliveries at our institution.

PACS numbers: 87.53.Kn, 87.53.Ly, 87.56.Da

## I. INTRODUCTION

**Figure 1:**
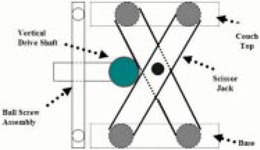
Schematic of the scissor jack elevator. The drive shaft is controlled by a motorized ball screw assembly. The elevation of the scissor jack is determined by the horizontal position of the drive shaft.

A tertiary micro multileaf collimator (MLC) system (3D Line, Reston, VA) was acquired for delivering conformal arc radiotherapy and radiosurgical treatments at our institution. The microMLC system is mounted on the block tray slot of a Clinac 6/100 accelerator (Varian, Palo Alto, CA). For the couch to deliver conformal arc treatments, we needed to improve the accuracy of its mechanical stability and setup. The original couch design was based on the scissor mechanism; that is, the vertical motion of the couch is driven by a scissor jack mounted on the base plane of the couch. The mechanism of the scissor action is illustrated in Fig. 1. The main advantage of the couch's scissor action is its ease of control and maintenance, since a single shaft is used to motorize the handlebars. However, a small displacement in the driving shaft will translate into substantial errors of the couch top. As a result, the isocenter alignment of the couch as defined by the radius of the spoke film locus is off by >3 mm over time. Further analysis shows that this error is caused mainly by the intercomponent motion of the scissor elevator and its link at the couch top. To overcome this problem, we designed several external stoppers to stabilize the couch. Our goal is to achieve couch isocenter alignment with an accuracy within 1 mm for implementing conformal arc deliveries.

## II. METHODS

**Figure 2:**
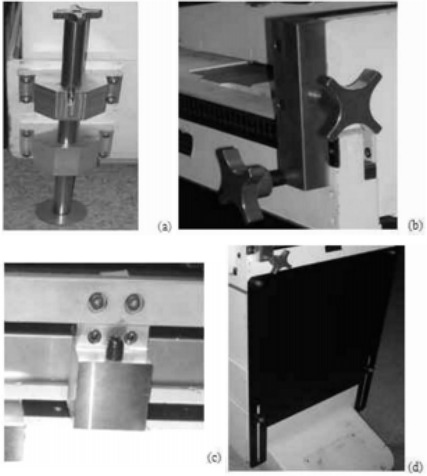
Illustration of the couch stabilizing devices: the latch rod (a); the corner stopper (b); the tap‐screw stopper along the track that moves longitudinally (c); and the double panel mounted at the end of the couch (d).

The major components of the couch are the following: (1) a longitudinal motion control carriage; (2) a lateral motion control carriage; and (3) a scissor elevator. The couch top is driven by a two‐roller support assembly through a chain motor.^(1)^ The longitudinal carriage is supported on a turntable connected to the driving motor in the floor pit. A scissor jack is mounted on the base plate of the turntable. Due to triangulation motion of the scissor jack, a 0.5‐mm shift in the lower pivot point potentially introduces a 5‐mm error of the couch top. We first stabilized the base plate of the couch by latching the couch base onto the concrete floor using a 5‐mm diameter aluminum rod. The latch is fitted into predrilled holes on the floor at 0°, 45°, 90°, 135°, and 270°. The positions of the holes are mechanically aligned using lasers, are countersunk, and fitted with the rod with an accuracy of 0.2 mm. As a result, the error in the couch rotation at the preset angles is reduced to <0.05°. The setup of the rod assembly is illustrated in Fig. 2.

To minimize the translational motion of the couch, 10 tap‐screw stoppers and 2 aluminum alignment panels were attached to the couch. Of the 10 stoppers, 2 were mounted on every corner of the couch top. On each corner, one stopper constrains the lateral motion, and another stopper constrains the longitudinal motion of the couch top. Two additional stoppers were placed along the track of the couch that moves longitudinally (Fig. 2). Two aluminum panels were mounted onto both the head and the foot end of the couch. The placement of the aluminum panels is also illustrated in Fig. 2. The purpose of the two panels is to connect the couch top to the immobilized base of the turntable to prevent pitch, roll, and yaw of the couch top. There are two slot tracks machined on each panel to adjust the height of the couch.

## III. RESULTS AND DISCUSSION

**Figure 3:**
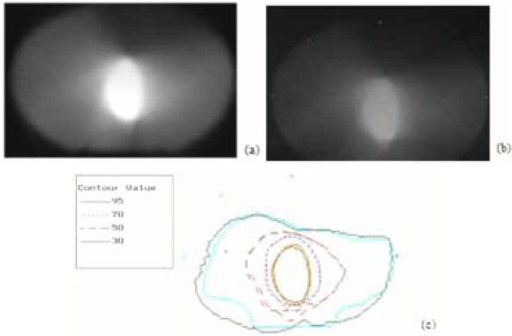
Results of the phantom measurements compared with the planned dose distributions: the planar dose distribution of the treatment plan calculations (a); the film measurements in the phantom (b); and the isodose overlay between the measurements and the calculations (c). The four fiducial markers are visible on the outer boundary of the film and isodose overlay plot.

We commissioned the couch for conformal arc beam deliveries with a microMLC system. For each treatment, mechanical checks and phantom measurements were performed for the couch. We measured the dose delivery using Kodak EDR2 film inside a cylindrical solid water phantom (CIRS, Norfolk, VA) to verify the accuracy of the treatment delivery. The film is pressed tightly in an axial plane of the phantom. There are also five internal fiducial markers that prick the film for referencing the isocenter alignment. The dose‐response curve of the film was calibrated using at least 10 points and analyzed using RIT software (RIT, Colorado Springs, CO). A result of the phantom measurements for an eight‐arc delivery is shown in Fig. 3. The eight arcs include one 40° sagittal and seven coplanar axial arcs of 20° each. During each arc delivery, the MLC field changes constantly when the gantry rotates. For different arcs, the gantry rotates with a different but plan‐optimized speed. The results of Fig. 3 show good agreement of isodose distributions between the measurements and the calculated treatment plans. We used 1‐mm and 3% criteria of the gamma function in RIT to measure the agreement between the isodose dose lines. The small discrepancy found at the posterior of the film was due to the supporting rails beneath the couch top. We also carried out mechanical alignment and spoke film checks at different couch angles and couch heights. The mechanical isocenter alignments were performed using the crosshair and lasers, using a precision‐alignment tool (Medtec, Orange City, IA). The spoke film analyses were carried out using RIT. We found that the couch deviated less than 1 mm for 16 repeated checks over a period of 7 months.

In summary, we demonstrated an effective method for commissioning a scissor‐action Varian couch for conformal arc treatments using a microMLC system. With several simple stabilization devices, we achieved the couch isocenter alignment to ±1 mm. Our method is cost‐effective: the entire project cost approximately $1000, including labor.

## ACKNOWLEDGMENT

Mr. Carlo Repeto machined the hardware for the project. This work is supported in part by a research grant from the Department of Defense and the S. G. Komen Breast Cancer Foundation.
